# Microorganisms in Plant Growth and Development: Roles in Abiotic Stress Tolerance and Secondary Metabolites Secretion

**DOI:** 10.3390/microorganisms10081528

**Published:** 2022-07-28

**Authors:** Ntombikhona Appear Koza, Afeez Adesina Adedayo, Olubukola Oluranti Babalola, Abidemi Paul Kappo

**Affiliations:** 1 Department of Biochemistry and Microbiology, University of Zululand, KwaDlangezwa 3886, South Africa; 2 Food Security and Safety Focus Area, Faculty of Natural and Agricultural Science, North-West University, Mmabatho 2735, South Africa; 3 Molecular Biophysics and Structural Biology Group, Department of Biochemistry, University of Johannesburg, Auckland Park 2006, South Africa

**Keywords:** abiotic stress, endophytes, plant biotechnology, plant microbiomes, secondary metabolites

## Abstract

Crops aimed at feeding an exponentially growing population are often exposed to a variety of harsh environmental factors. Although plants have evolved ways of adjusting their metabolism and some have also been engineered to tolerate stressful environments, there is still a shortage of food supply. An alternative approach is to explore the possibility of using rhizosphere microorganisms in the mitigation of abiotic stress and hopefully improve food production. Several studies have shown that rhizobacteria and mycorrhizae organisms can help improve stress tolerance by enhancing plant growth; stimulating the production of phytohormones, siderophores, and solubilizing phosphates; lowering ethylene levels; and upregulating the expression of dehydration response and antioxidant genes. This article shows the secretion of secondary metabolites as an additional mechanism employed by microorganisms against abiotic stress. The understanding of these mechanisms will help improve the efficacy of plant-growth-promoting microorganisms.

## 1. Introduction

A significant problem for humanity in the 21st century is the production of sufficient food for an exponentially growing population [1]. This challenge is compounded by a decline of arable farmlands brought about by human occupancy, soil degradation, and a wide variety of environmental factors, including flooding, drought, salinity, temperature (extreme heat and cold), and pollution by heavy metals [2]. Plants, being sessile, have evolved ways through which they perceive and respond adaptively to stress conditions, and some of the most common and extensively documented include the production of osmolytes, alteration of water movement, and scavenging of reactive oxygen species [3].

Plant biotechnological methods have been used to develop crop varieties that can resist disease, tolerate stressful conditions, and above all, provide better nutritional value [4]. These methods involve the selection and introduction of desirable traits from one plant to another by means of conventional breeding or genetic engineering [4]. Although these approaches can potentially produce genetically modified crops/plants capable of withstanding stressful conditions, their affordability and availability to only developed nations is a limiting factor that still needs to be addressed. Microorganisms are mainly considered harmful due to their disease-causing properties [5]. However, some are beneficial in agriculture, and are now being used in the production of sustainable food crops [5,6]. Beneficial microorganisms have been shown to play a role in atmospheric nitrogen fixation, organic wastes and residues decomposition, detoxification of pesticides, suppression of plant diseases and soil-borne pathogens, enhancement of nutrient cycling, and production of bioactive compounds such as vitamins, hormones, and enzymes that upregulate plant growth [7,8]. Examples of microorganisms that have been used to limit abiotic stress include plant-growth-promoting rhizobacteria (PGPR) and plant-growth-promoting fungi (PGPF) [3,9,10].

Studies have shown that beneficial microbes suppress abiotic stress using various strategies including growth promotion by stimulating the production of phytohormones, siderophores, and solubilizing phosphates; lowering ethylene levels; and upregulating the expression of dehydration response and antioxidant genes [2]. Kushwaha, et al. [11] reported that root-colonizing bacteria produce phytohormones that alleviated salinity-induced dormancy and elicited seedling growth. Moreover, Kumar, et al. [12] showed that plant-growth-promoting bacteria *Pseudomonas* sp. and *Bacillus* promoted growth in stressed plants by producing indole acetic acid (IAA), siderophores, and solubilizing phosphates. Furthermore, some of these plant-growth-promoting microorganisms contain 1-aminocyclopropane-1-carboxylase (ACC) deaminase, an enzyme that cleaves plants’ ethylene precursor ACC, thereby inhibiting ethylene synthesis in stressed plants [13,14]. Lowered ethylene levels resulted in root growth and improved the survival of stressed plants [15]. While it was clear that ACC-deaminase-containing microorganisms and phytohormones helped alleviate various stresses in plants, it has been documented that other microorganisms employ different strategies to confer stress tolerance to plants [16]. 

Kaushal [17] showed that rhizobacterium *Paenibacillus polymyxa* enhanced drought stress tolerance by augmenting the expression of the EARLY RESPONSIVE TO DEHYDRATION 15 (ERD15) gene. More so, Zhang, et al. [18] showed that *Arbuscular mycorrhizal* (AM) fungus improved plant growth, biomass production, and osmotic adjustment (by increasing the uptake of K^+^, Ca^2+^, and Mg^2+^ ions) of drought-stressed tangerine (*Citrus tangerine*). Some PGPR strains have also been shown to improve stress tolerance by producing antioxidants that degrade reactive oxygen species (ROS) [19]. AM fungal hyphae can reach soil pores that are not reachable by root hairs, assimilating water and nutrients that cannot be assessed by non-AM plants [20], thereby promoting plant growth by changing the plant–water relationship, resulting in improved efficiency of water use and, consequently, promoting the yield of the crop [21]. Beneficial strains of mycorrhizal are occasionally employed in consortium with PGPR for enhanced efficiency in plant growth promotion, making mycorrhizal symbiosis utilization more attractive for sustainable agricultural productivity [21].

Although most reports show that abiotic stress relief by microorganisms is through the activation of primary metabolisms such as plant growth, nutrient uptake, photosynthesis, and antioxidant enzymes, there is evidence implicating the involvement of secondary metabolites [22]. Some secondary metabolites such as flavonoids, phytoalexins, phenylpropanoids, and carotenoids have been documented in stressed plants inoculated with microorganisms [23,24]. These secondary metabolites help plants tolerate abiotic stress by acting as antioxidants that scavenge ROS [25]. Both fungi and bacteria have been commonly implicated in the induction of secondary metabolites in stressed plants. 

The soil remains an unpredictable environment due to numerous factors present therein; hence, there is difficulty in explaining the dynamic interactions that exist within. These factors include soil type, climatic condition, pH, temperature, an abundance of other microbes in the rhizosphere, and the type of plants growing in a particular soil, as well as chemical fertilization of the soil, which affects the colonization of plant roots [26]. Even though there seems to be great variability in the colonization of plants by microorganisms, there is a possibility that positive effects may be obtained. It has been shown that when microbes are isolated from harsh environmental stress conditions, the alleviation of abiotic stress is possible [27,28]. Hence, this review article discusses the beneficial effects of microbes on abiotically stressed plants and highlights the microbial strains that are effective at reducing abiotic stress effects to deploy them under extreme environmental conditions.

## 2. Abiotic Stress

Abiotic stress is an environmental factor that limits plant growth and metabolism [29]. It has been estimated that abiotic stresses reduce yields and production of major food and cash crops by more than 50% [30]. There are two classes of abiotic stresses, namely above and below ground abiotic stresses. Atmospheric-induced abiotic stresses are those that originate from the atmosphere, whereas edaphic abiotic stresses occur in the soil [31]. Abiotic stresses of atmospheric origin are common in areas where climate variability and precipitation patterns change with longer periods of drought intermixed with spells of heavy rainfall [32]. Abiotic stresses of edaphic origin, on the other hand, may arise from anthropogenic activities involving the use of brackish water and sewage water for irrigation, sewage sludge for fertilization, and inorganic chemicals for fumigation [33]. This problem is often exacerbated by improper waste disposal methods, weathering of native rocks, and poor cultural practices, which have rendered huge expanse of lands unarable for crop production [10,23]. If these stresses are not properly managed they will fluctuate significantly in intensity and duration, thereby exhibiting a net effect on global agriculture [3].

### 2.1. Effects of Abiotic Stress on Plants

**Figure 1:**
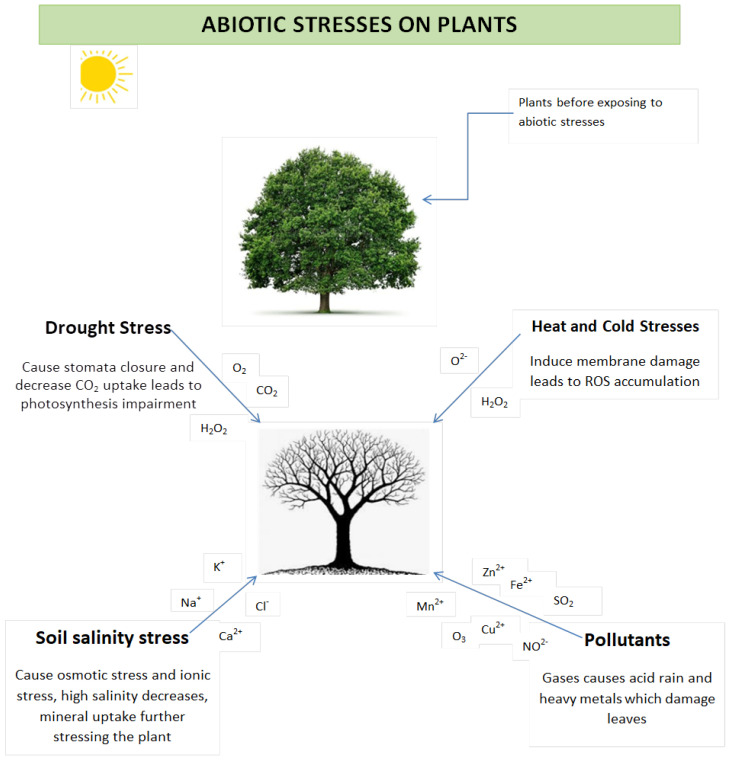
Effect of abiotic stress on plant morphology, physiology, and biochemistry. Abiotic factors have negative effects on plants’ growth, quantity, and quality; these effects can reduce plant productivity and permanently damages the plants when exposed for a longer period. High concentrations of sodium (Na), Chloride (Cl), and potassium (K) cause ion cytotoxicity in plants; the closure of stomata inhibit the exchange of oxygen (O_2_) and carbon dioxide (CO_2_). Plants leaves have been damaged by air pollutants such nitrogen oxide (NO_2_), ozone (O_3_), and sulfur dioxide (SO_2_) and soil pollutants such as mercury, iron (Fe), zinc (Zn), and copper (Cu) (UN-Oceans 2008). Lastly, all abiotic stresses leads to the overproduction of ROS, such as superoxide anions (O^2−^) and hydrogen peroxide (H_2_O_2_), which are very reactive and damage or kill the plant.

Abiotic stresses are known to trigger a series of molecular events leading to changes in the morphology, physiology, and biochemistry of plants [34]. This review paid a closer look at the effects of drought, soil salinity, soil pollutant, and extreme temperature (Figure 1) on plant morphology, physiology, and biochemistry. Drought, a meteorological term used to define a period without substantial rainfall, affects 26% of arable lands and it is considered a major limiting factor among other abiotic stresses [35]. It constrains yield stability and crop production in arid and semiarid areas [36]. It is a meteorological term used to define a period without substantial rainfall [37]. It has been reported that one-third of the world’s population resides in areas where water is scarce [35]. Drought also depends on evaporative demands and moisture-storing capacity of the soil in the area [38]. Drought stress in plants does not only affect yield and crop productivity, but it also restricts plants’ ability to assimilate soil nutrients and water; and inhibit photosynthesis; reduces endogenous cytokinin levels, while increasing that of abscisic acid in the leaves, thereby causing the stomata to close [39].

To prevent water loss, plants respond by closing the stomata early to maintain the water level inside the plants. However, this response has negative effects during gaseous exchange such as the plants’ intake of water vapor, carbon dioxide (CO_2_), and oxygen (O_2_) from the air using their leaves [40]. The decrease in CO_2_ for instance affects photosynthesis since CO_2_ limitation causes a decrease in photosynthetic fixation in plants [25]. Furthermore, stomata closure disrupts the photosynthetic machinery of plants as the oxygen level is reduced by photosystem I, resulting in the production of superoxide (O^2−^) and H_2_O_2_, which hastens the water–water cycle [2]. 

Stomatal closure also interrupts transpiration, which is an important barrier between gas and water loss in the soil [40]. Moreover, drought stress in plants also leads to molecular and biochemical changes. Water deficit strongly affects activities that are very sensitive to water limitation such as cell wall formation, cell expansion, maintenance of turgor pressure, and vital metabolic functions [41]. Under drought stress, plants undergo different structural modifications such as the reduction in height and size of leaves, stems, shoots, and roots [42]. A study by Yordanov and colleagues (2000) reported a close correlation between “drought resistance” and “dehydrated accumulation” in *Populus* spp. and wheat (*Triticum* spp.) [41]. Furthermore, severe drought in plants can also lead to oxidative stress, which affects biomolecules’ structure and functions. Oxidative stress causes the oxidation of nucleic acids, protein, membrane lipid peroxidation, and enzyme inhibition [43].

Mineral toxicity or deficiency is the second major limiting factor with enormous effects on agricultural productivity. Among mineral toxicity factors, salinity is widespread and is estimated to affect 10% of the world’s land surface, especially in irrigated areas. If left unattended, increasing salinity is predicted to have a devastating effect on about 50% of the land devoted to crop production by 2050 [32]. Salinity affects plants in three ways: Restriction of water uptake hinders the nutrient absorption mechanism and the induction of ion cytotoxicity. Firstly, salinity restricts water uptake by impairing the ability of plants to absorb water from the soil leading to lower soil water potential. Secondly, it interferes with the plant’s nutrient absorption mechanism, thereby causing a nutrient imbalance in plants [43].

Under long-term salt stress, ionic components in plants are elevated, thereby increasing ion concentration, which is very poisonous to plants, leading to ion cytotoxicity [44]. Ion cytotoxicity-associated leaf death, for instance, reduces the photosynthetic capacity of plants by minimizing carbohydrates’ uptake by young leaves, which is necessary for growth, thus reducing the growth rate of the plants in totality [40]. High salt concentration in plants leads to toxic Na^+^ concentration and membrane lipid peroxidation; the toxicity can seriously affect the concentration of both Cl^−^ and Na^+^. Increased Na^+^ concentration can lead to the inhibition of potassium (K^+^) ion uptake, which plays an important role in plants’ physiological processes such as protein synthesis, osmotic regulation, and enzyme activity. Deficiency in K^+^ leads to ion imbalance, loss of protein function, and conformational changes in biochemical reactions in plants [45]. Finally, salinity has been shown to impose toxic effects such as protein synthesis inhibition, cell organelle damage, disruption of enzyme structure architecture, and the uncoupling of photosynthesis and respiration in plants [45]. Plants under excessive salts stress show structural phenotypes such as yellow spots (necrosis) on leaf edges; decreased root and height growth, root, shoot, and stem length, and bud formation; and degenerate fruit color and flavor [30]. These effects are similar to those imposed by drought stress on plants, and as a result, retards growth and reduces yield [46].

Soil contamination by heavy metals is another form of mineral toxicity with physiological relevance in plants. Anomalous high concentrations of heavy metals in soils are due to mining, parental rocks, and metal processing [47]. The commonly encountered effects of heavy metal toxicity in plants are demonstrated by limited photosynthetic rate, increased heavy metal uptake, and the disruption of root hydraulic conductivity via apoplastic and symplastic pathways [48]. The soil is composed of different heavy metals such as mercury, arsenic, nickel, copper, chromium, lead, cadmium, zinc, and iron, which play an important biological role in plant growth and development. Anthropogenic activities and releases from natural resources in the form of continental dust and volcanic activities remain the major source of heavy metals [49]. A high concentration of heavy metals strongly affects plant growth by causing toxic effects that hinder nutrient uptake by the plants, leading to the impairment of membrane integrity and also affecting enzyme activity of the plants’ cells. The toxicity of heavy metals can lead to the production of ROS and oxidative stress, which further inhibits enzyme activity and blocks metabolite functional groups in plant cells. A high concentration of iron (Fe^2+^) in plant cells is harmful as this metal highly reacts with oxygen to form ROS [50]. Overstimulation of ROS results in oxidative stress and consequently damages plant organelles leading to the eventual death of the plant [49].

Another major limiting factor to crop production are temperature stress. When temperatures fall below zero, plants are exposed to various freezing stress. These include osmotic injury, desiccation, loss of stomatal control, reduced efficiency of the photosynthetic apparatus through pigment modification, the decline in fluorescence, and impaired chloroplast apparatus. Plants are also sensitive to temperature fluctuations [51]. It has been shown that a sudden increase in the ambient temperature by 5–7 °C creates heat stress in plants. These temperatures have been reported to disrupt photosynthesis, reduce plant water, interfere with flowering/fruiting, and attract pests and diseases [52]. Temperature-related effects on plants have been reported in tropical and temperate regions of the world. Chilling stress, for example, is a form of temperature stress, a nonfreezing stress that plants experience when the temperature reduces from 15 to 0 °C [53]. Regarding sensitive plants, this stress causes numerous injuries such as mechanical constraints (membrane integrity), osmotic stress, increased respiration and ethylene production, loss of chlorophyll and reduction in photosynthesis, uneven ripening, disease susceptibility, water soaking, and surface pitting. Figure 1 depicts the effect of abiotic stress on plant morphology, physiology, and biochemistry.

### 2.2. Bacteria in Abiotic Stress Tolerance 

**Figure 2:**
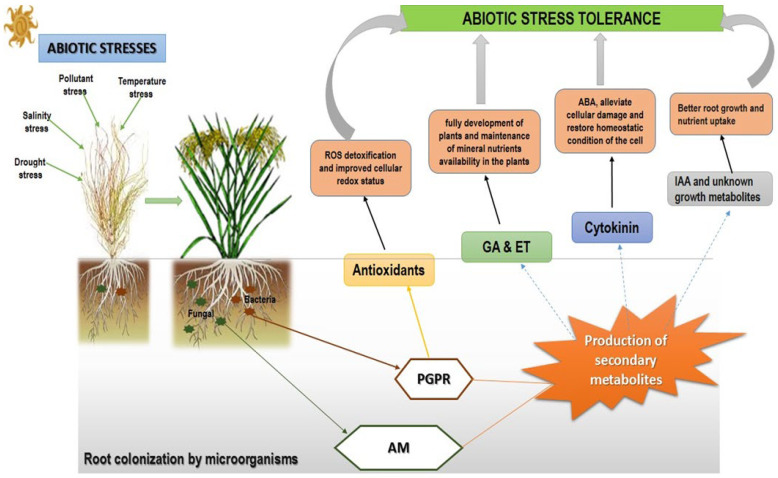
Proposed model of PGPR and AM against abiotic stress in plants: Microorganisms colonize roots by using different mechanisms such as triggering an ionic and osmotic response. Microbial inoculation, such as PGPR indicated in brown on the plants’ roots, AM, and EM, indicated with a green solid arrow, are often reported to protect plants against water drought stress, thus increasing dehydration intolerance. PGPR, AM, and EM have shown to produce antioxidant activities (yellow arrow) such as catalase (CAT), peroxidase (POD), and superoxide dismutase (SOD), which improves osmotic adjustment in plants and secondary metabolites (broken blue arrows) such as cytokinin (CKs), gibberellins (GAs), ethylene (ET), and auxins (IAA), which elicit stress tolerance in plants, resulting in root surface area, root length, and the number of root tips. Antioxidants and secondary metabolites help in enhancing the uptake of nutrients from the soil by the roots to produce a high yield of plant crops [58].

Of all the microorganisms found in the soil, bacteria are by far the most common [54]; hence, some of them found colonizing the roots of plants are referred to as PGPR. Numerous bacterial families have been shown to be involved in the improvement of plant growth under stressful conditions (Figure 2). In drought-stressed plants, it has been shown that PGPR such as *Paenibacillus polymyxa, Achromobacter piechaudi, Azospirillum brasilense, Pseudomonas* sp., *Burkholderia, Arthrobacter, Microccocus luteus*, and *Bacillus* enhanced the drought tolerance of *Arabidopsis thaliana* [16], pepper, tomato [13,22], wheat [55] and maize [56] plants. Some of these bacterial-induced tolerances have been associated with an increase in mRNA transcription of the drought-response gene EARLY RESPONSIVE TO DEHYDRATION 15 (ERD15), the production of 1-aminocycloropropane-1-carboxylic acid (ACC) deaminase, stronger proline synthesis, and an improvement of relative and absolute water content [25]. Kasim and colleagues (2013) reported that priming with two PGPR strains, *Bacillus amyloliquefaciens* 5113 and *Azospirillum brasilense* NO40, attenuated drought-induced stress results in wheat plants by increasing the activity of antioxidant enzymes (glutathione peroxidases (GPXs)) against ROS [55]. These findings supported the potential of the use of PGPR in controlling drought stress and increasing crop production. Carlson, et al. [57] proposed that the physical and chemical changes that PGPR elicit to protect plants from abiotic stress be referred to as “induced systemic tolerance” (IST).

IST via PGPR has also been documented in the mitigation of salt stress in various plants. In lettuce seeds, *Azospirillum* inoculation improved germination and vegetative growth when exposed to NaCl [57]. Some studies have also highlighted the relevance of decreasing endogenous ethylene levels as bacterial-mediated tolerance to salt stress [59]. ACC deaminase-containing bacteria such as *Achromobacter piechaudii* ARV8, *Pseudomonas fluorescens,* and *Bacillus* sp. have been shown to improve salt tolerance in the growth of tomato seedlings, groundnuts, and the salinity-induced osmotic stress of pepper [55]. *P. fluorescens* has also been shown to improve tolerance by decreasing the synthesis of osmolytes and the production of salt stress proteins such as osmotins and dehydrins [56]. Kerbab, et al. [60] found that in some instances PGPRs are effective at reducing salt stress effects when inoculated in combination with other microbes. For example, *Pseudomonas mendocina*, alone or in combination with an AM fungus such as *Glomus intraradices* or *Glomus mosseae,* synergistically improves growth, nutrient uptake, and other physiological activities of salt-stressed *Lactuca sativa*. 

PGPR have long been shown to also combat the effects of extreme temperatures. Keswani, et al. [61] found that the bacterial strain *Pseudomonas putida* GR 12-2 promoted growth and root elongation of canola when exposed to 5 °C during both springs and winter. Other studies showed that bacterial strains such as *Serratia liquefaciens*, *Serratia proteamaculans,* and *Bradyrhizobium japonicum* had beneficial effects on the growth and physiology of soybeans grown under suboptimal root zone temperatures [61]. Furthermore, it was observed that the inoculation of potatoes with rhizobacteria such as *Burkholderia phytofirmans* PsJN played an adaptive role in heat stress. The same bacteria, *Burkholderia phytofirmans* PsJN, when inoculated with cold-stressed grapevine *Vitis vinifera*, lowered biomass reduction and electrolyte leakage from membranes [62]. The bacterial “IST” to cold stress includes increasing sugar levels, upregulating the expression of antifreeze-containing proteins, and increasing the levels of proline content [63]. 

PGPR has also been implicated in the alleviation of heavy metal stress. Pseudomonas brassicacearum strains were isolated and characterized, and *Pseudomonas marginalis*, *Pseudomonas oryzihabitans*, *Pseudomonas putida*, *Pseudomonas tolaasii* ACC23, *Alcaligenes xylosoxidans, Alkaligenes* sp. ZN4, *Variovorax paradoxus, Bacillus pumilus,* and *Rhodococcus* sp. can be used to counteract the effect of cadmium stress on plants. They found that these bacterial strains improved the growth of the metal-accumulating plant Brassica juncea [64]. It has also been documented that the PGPR strains A3 and S32 promoted the growth of Brassica juncea under chromium stress conditions [12]. Furthermore, the bacterial strain *Kluyvera ascorbata* SUD165 protected canola seedlings from nickel toxicity [65]. The “IST” elicited by these bacterial strains against heavy metal stress was associated with ACC deaminase activity and production of siderophores and auxins. Above all, these inoculants may be useful in phytoremediation. Regardless of the precise mechanisms bacteria use against environmental stresses, there is a possibility that plant-growth-promoting bacteria may adapt plants to these environmental stresses. This will help stabilize and revegetate abandoned lands due to abiotic stress, resulting in a concomitant increase in food production.

### 2.3. Differences in the Mechanisms of Overcoming Stress by Plants under the Influence of Rhizospheric and Endophytic Microorganisms 

Microorganisms employ biochemical and molecular mechanisms through the interaction of plants and microorganisms that assist in modifying the negative effect of abiotic stresses on the growth of plants [66]. Phytohormones such as auxins, cytokinins, and gibberellins carry out a special function in changing the morphology of the roots of the plant [67], thereby modifying the adaptation of plants and tolerating certain abiotic stresses such as drought, heavy metal, nutrient deficiency, and salinity. The production of hormones such as auxins promotes cell elongation in plants root, as well as the growth of lateral roots that contribute a positive effect to the assimilation of nutrients and water acquisition by the plants. The negative effects of abiotic stress were mitigated by PGPR by induced systemic tolerance (IST), which includes the following: (i) production of phytohormones such as Auxins, abscisic acid (ABA), and cytokinins; (ii) secretion of various antioxidants such as (Superoxide dismutase) SOD, peroxidase (POD), APX that catalyzes the transformation of H_2_O_2_ into H_2_O, catalase (CAT) enzyme that improve the growth of plants, glutathione reductase (GR) that converts oxidized glutathione (GSSG) into reduced glutathione (GSH) through the ascorbate–glutathione cycle; and (iii) degradation of the ethylene precursor ACC by bacterial ACC deaminase [68]. Misra and Chauhan [69] reported how plants were inoculated with PGPR containing the ACC deaminase enzyme that has the potential of mitigating abiotic stresses by controlling the production of ethylene through metabolizing ACC into alpha-ketobutyrate and ammonia. Microorganisms have the potential to improve plant growth under abiotic stress conditions by promoting the production of low-molecular-weight osmolytes, such as glycinebetaine, proline, and other amino acids, mineral phosphate solubilization, nitrogen fixation, organic acids, and producing key enzymes such as ACC-deaminase, chitinase and glucanase [70]. These microorganisms are also involved in the promotion of tolerance of heavy metals by transporting them across the cell membrane, accumulation on cell walls (intra and extracellular), redox reactions, and production of complexes [71]. 

Endophytes involve a symbiotic association within the plant and possess the potential to inhabit the internal tissues of the plant through the leaf, root, seed, and stem of a host plant. They are also involved in nitrogen fixation, phytohormones secretion, and acquisition of nutrients, thereby improving the growth of the plant. Root exudates are known to be produced by plants that act as the source of energy for endophytic microbes associated with them [72]. While endophytic organisms were colonizing the plants at an early stage, there is the production of exopolysaccharides (EPS) from bacterial cells that alleviate its attachment to the surface of the root and likewise prevent bacterial cells from oxidative damage [73]. Arbuscular mycorrhizal fungi are well-reported fungi that promote nutrient assimilation in plants and tolerate a lot of conditions of abiotic stress [74]. The AMF activates a mutualistic relationship with its host plant, controlling the growth of crop plants. The network of mycelial of AMF widens below the roots of the crop plant, therefore improving nutritious assimilation. The common mycorrhizal network (CMN) has an intense consequence on the fungal-mediated transport of nitrogen (N) and phosphorus (P) to plants, and thereby aids the growth of plants under stressful environmental conditions [75]. 

### 2.4. Pesticides Stress on Crop Plants

The effect of polycyclic aromatic hydrocarbon fluoranthene has the effect of organic pollutant stress on the growth of seeds and roots of *Zea mays* and *Pisum sativum*. The germination of the seed was suppressed in maize and pea [76]. Pesticides can negatively affect cellular metabolism when there is an increase in reactive oxygen species (ROS) levels, biochemistry, and the physiological machinery of plants [77]. Hatamleh, et al. [77] reported how various pesticides including diazinon (DIZN), imidacloprid (IMID), and mancozeb (MNZB) have been applied to tomato plants. The greater doses of pesticides applied increase ROS levels and stimulate the damage of membrane by stimulation of thiobarbituric acid reactive substances (TBARS), thereby increasing cell injury. To avoid pesticide-induced oxidative stress, plants affected with greater pesticide dosages reveal a high level of antioxidant levels. Increased levels of pesticides resulted in modifications in the membrane of the mitochondrion and cause the death of cells in the plant roots.

## 3. Fungi in Abiotic Stress Tolerance

### 3.1. Arbuscular Mycorrhizal Fungi in Alleviation of Abiotic Stress

In addition to bacterial involvement in stress tolerance, fungi have also been implicated in adapting plants to various habitats, including those that are affected by abiotic stresses such as salinity, chilling, drought, heat, toxic metals, and flooding [78]. Two types of fungi are involved in stress tolerance, these include arbuscular mycorrhiza (AM) and ectomycorrhiza (EM) fungi. AM live inside the host plant without causing any harm and have been reported to evoke various stress tolerances. Poveda, et al. [79] showed that abiotic stress tolerance could be induced by exploiting the abundant endophytic arbuscular mycorrhizal fungi, which exist in reciprocally beneficial relationships with about 80% of plants. Endophytes that confer tolerance are further divided into Class 1 and Class 2 [74]. Class 1 endophytic fungi are known to have a narrow range of hosts because their colonization is limited to shoots, stems, and rhizomes. Class 2 endophytes, on the other hand, confer habitat-specific stress tolerance to monocots and eudicots [80]. Thus, they have a broad range of hosts in which they confer habitat adaptation benefits. It has been reported that both endophytes improve tolerance to abiotic stress [81]. Endophytes that have been reported to confer tolerance to abiotic stress include *Curvularia protuberata, Fusarium culmorum, Piriformospora indica, Phoma glomerata* LWL2, *Penicillium sp.* LWL3, *Paecilomyces formosus* LHL10, *Neotyphodium lolii,* and *Trichoderma* [82]. These fungi confer stress tolerance by influencing the nutritional, physiological, and biochemical properties of the host plant. These include overproduction of siderophores, reduction in ethylene synthesis via ACC-deaminase production, enhancement of antioxidant activity, inhibition of sodium uptake by increasing uptake of electrolytes such as K^+^, accumulation of proline, and improving root water uptake and enhancing antioxidant capacity [77]. 

It has been documented that AM can promote salt tolerance using various mechanisms, which include adjusting the rate of K^+^/Na^+^ in the plant cell, transfer of ion salts to the vacuole, production of growth hormones, and improvement of rhizospheric and soil conditions, as well as improvement of photosynthetic efficiency or water use efficiency [83]. Moreover, AM ameliorates salt stress effects by increasing sugar and electrolyte concentrations, and hence functions as osmoregulators [84]. In another study, AM induced tolerance to salt stress by upregulating antioxidant capacity by activating the plant glutathione–ascorbate cycle. AM symbiosis has also been demonstrated to improve the salt resistance of plants such as maize, clover, tomato, cucumber, and lettuce [84]. 

In drought-stressed plants, AM-induced tolerance has been shown as a common feature of abiotic stress tolerance [85]. Investigations showed that on *Leymus chinensis* (C3) and *Hemarthria altissima* (C4), the production of grasses was modified intensely by water stress that increased the biomass of the plant (58%), intrinsic efficacy of water usage (15%), stomatal conductance (38%), photosynthetic rate (63%), and SOD activity (45%), with a decrease in the concentration of malondialdehyde by 32% of *Leymus chinensis* under mild (30%) and moderate (50%) drought stresses [2,20].

It has been shown that in some species of *Festuca*, infection with *Neotyphodeum* endophytic fungi improved growth and biomass production under drought stress conditions [82]. Furthermore, the effects of endophytic fungi on the drought tolerance and recovery of *Lolium perenne* have also been shown to be beneficial [81]. In other plants, endophytes alleviated drought stress by altering physical, nutritional, physiological, and cellular processes [86]. These include the improvement of germination and water uptake (via aquaporins), reducing transpiration (overproduction of abscisic acid leading to stomatal closure), improvement of plants’ resistance to drought, accumulation of proline, and improving antioxidant activities by reducing damage caused by free radicals generated during drought [25]. Plants whose drought tolerance has been shown to be improved by mycorrhizal inoculation include wheat (*Triticum aestivum*), coriander (*Coriandrum sativum* L.), basil (*Ocimum basilicum*), grapes (*Vitis vinifera*), onion (*Allium cepa* L.), and sorghum (*Sorghum bicolor*) [85]. 

AM has also been reported to boost plant tolerance to temperature fluctuations. In particular, AM *Glomus mosseae* has been shown to alleviate the damage caused by chilling stress on tomato plants (*Lycopersicon esculentum* cv Zhongzha105) by reducing membrane lipid peroxidation, while increasing the level of photosynthetic pigments, accumulation of osmotic adjustment compounds, and antioxidant enzyme activity [87]. In heat-stressed plants, AM confers stress tolerance by increasing the levels of phytohormones to prevent premature plant senescence and enhance the level of secondary metabolites (proline and anthocyanins) [88]. In chilling stress, AM has been shown to attenuate membrane lipid peroxidation and plasma membrane permeability, thereby increasing osmolyte accumulation, activating antioxidant enzymes, and improving photosynthetic activity [89].

In metal-toxic soils, AM also plays an important role in heavy metal tolerance. For example, AM inoculation has been shown to enhance growth in tomato seedlings, canola, maize, and rice grown in heavy-metal-contaminated soils by minimizing the uptake of toxic metals; enhancing the uptake of essential nutrients from the soil; and the production of glycoproteins, glomalin, and cell wall chitin, which complexes to heavy metals [87]. Furthermore, AM improves the performance of heavy-metal-stressed plants by increasing the activity of antioxidant enzymes and the subsequent accumulation of soluble amino acids. AM ecotypes, used in the alleviation of heavy metal stress, are those isolated from soils contaminated with high concentrations of heavy metals, and they include *Glomus mossae, Glomus claroideum,* *Viola calaminaria,* and *Aspergillus niger* [90].

### 3.2. Ectomycorrhiza in the Alleviation of Abiotic Stress 

While most studies have been conducted on the amelioration of abiotic stress by endomycorrhizal fungi AM, there is little information available on ectomycorrhizal involvement [88]. The reason for this is that endomycorrhizae prevail on most herbaceous and woody species [91]. However, the ability of ectomycorrhizal fungi to alleviate abiotic stress has been demonstrated. Ectomycorrhizal fungi such as *Hebeloma crustuliniforme, Laccaria bicolor,* and *Laccaria laccata* have been reported to induce salt tolerance in white spruce (*Picea glauca*), jack pine (*Pinus banksiana*), and loblolly pine (*Pinus Teada*), respectively [92]. Basidiomycetes such as *Hebeloma cylindrosporum* and *Pinus pinaster* have also been identified as one of the ectomycorrhizal fungi that attenuate the detrimental effects of salt stress by increasing biomass production, water conductance, and limiting the loading of Na^+^ into the xylem while increasing that of K^+^ [93]. Furthermore, it was shown that inoculation of salt-stressed jack pine (*Pinus banksiana*) with ascomycetes *Cenococcum geophilum* improved tolerance and helped in the afforestation of abandoned farmlands [92].

Ectomycorrhiza has also been implicated in the alleviation of drought stress. It has been hypothesized that ectomycorrhiza improves drought stress effects by enhancing osmotic adjustment, enhancing tissue elasticity, and regulating gene expression [94]. While it is often assumed that ectomycorrhizal fungi die when soils dry out, studies have shown that some ectomycorrhizal species persist in dry soils and characteristically confer tolerance to drought stress in plants [95]. Boyle and Hellenbrand (1991) showed that when inoculated with ectomycorrhizal fungi, jack pine (*Pinus bankisiana*) and black spruce (*Picea mariana*) performed better when subjected to water stress. The seedling performance of these plants, when measured via their photosynthetic rate, shoot, and root growth, is better [96]. Martins and colleagues (1996) showed that mycorrhization of the European chestnut (*Castenea sativa*), an economically important forest tree with ectomycorrhizal fungi such as *Amanita muscaria, Laccaria laccata, Piloderma croceum*, and *Pisolithus tinctorius*, increased resistance to water stress [96].

Ectomycorrhiza has also been shown to mediate heavy metal stress tolerance. Ma and colleagues (2014) showed ectomycorrhiza, *Paxillus involutus*, enhanced cadmium tolerance by increasing detoxification into vacuoles, thereby improving the nutritional and carbohydrate status of *Populus canescence* [97]. The hyphae of ectomycorrhizal fungus, *Suillus bovinus*, are responsible for the accumulation of heavy metals such as zinc, which could either be deposited in the fungal cell wall or sequestered into the vacuole of the fungi [98]. 

Ectomycorrhiza has also imparted beneficial protection to plants exposed to low temperatures. Landhausser and coworkers (2002) showed that the ectomycorrhizal inoculation of aspen (*Populus tremuloides*) and white spruce (*Picea glauca*) seedlings at low soil temperatures of 4 °C and 8 °C improved tolerance [98]. Under heat stress conditions of temperate and boreal forests, ectomycorrhiza increased the performance of the cork oak tree (*Populus tinctorius*) by increasing leaf area, nitrogen acquisition, photosynthesis capacity, and water-use efficiency. Ectomycorrhizal fungi that induce protection against heat stress include *Lacarria bicolar* and *Tuber melanosporum* [99].

## 4. Abiotic Stress Tolerance Induction via the Accumulation of Secondary Metabolites by Microorganisms

The ability of microorganisms to protect plants against abiotic stress cannot only be attributed to the improvement of primary metabolic activities such as plant growth, nutrient uptake, photosynthesis, and antioxidant production but can also be associated with the production of secondary metabolites [100]. Secondary metabolites (SM) are chemical compounds that have no fundamental role in the growth and development of plants but perform specific functions under a given set of conditions [101]. Such functions include their involvement as active and potent defense agents against pathogens and herbivorous animals. Even though secondary metabolites play a vital defense role, they were once viewed as waste products with little importance in plant growth and development [22]. Recently, this view has now become irrelevant as many secondary metabolites not only defend plants from pathogens and herbivorous organisms but also protect plants from environmental stress [102]. A common consequence of environmental stress in plants is the increased production of ROS, which may cause oxidative stress that is characterized by the peroxidation of proteins, membrane lipids, and nucleic acids [103]. 

It has been reported that inoculation of environmentally stressed plants with microorganisms enhances the production of secondary metabolites. These compounds have been reported to add taste, odor, and color to plants and also play a significant role in signaling, defense against herbivore, and infection with fungi, bacteria, or yeast [104]. The biological activities of SM can be explained based on their structural diversity and biosynthetic origin. Both biotic and abiotic stress conditions are known to hinder plants growth and development under normal physiological conditions with a concomitant reduction in yield. Therefore, to maintain proper growth and development, as well as be protected against environmental stresses, the upregulation of SM becomes a prominent survival mechanism against deleterious conditions such as pathogens attack, heavy metals, extreme temperature, light intensity, salinity, toxic gases, drought, pollutants, and nutrient deficiency. The biosynthesis of SM plays a very important role in protecting plants against stresses, but more importantly, influences plant growth and productivity [78].

Several groups of SM have been reported such as alkaloids, terpenes, and phenylpropanoids. Alkaloids are nitrogen-containing SM with a family of more than 12,000 compounds with low molecular weights [105]. The biosynthesis of alkaloids was more observed when plants are exposed to high-temperature conditions and begins with reactions involving amino acids such as tryptophan, tyrosine, ornithine, and phenylalanine [106]. The family of alkaloids includes purine alkaloids, pyridine alkaloids, tropane alkaloids, benzylisoquinoline alkaloids, and terpenoid indole alkaloids, which account for about 3000 compounds [107]. The synthesis of terpenoid indole alkaloids requires tryptophan, which is known to improve plant growth and development, as well as protect plants against virus, bacteria, and fungi infestations [108]. Alkaloid compounds from microbial endophytes are known to possess antiviral, antifungal, antibacterial, and insecticidal properties [109]. Furthermore, pyridine alkaloids from *Nicotiana* spp. were reported to have a repellent effect on pollinators and reduce larval survival of honey bees at high concentrations [110]; these alkaloids and some other compounds provide plant protection against insect herbivores. Purine alkaloids in *Coffea* spp. and *Citrus* spp. have been shown to enhance food odor, recruit caffeinated food sources, and increase pollination of caffeinated flowers [111].

Natural compounds regarded as the largest group are terpenes that are synthesized by terpene synthases. About 40,000 structures of natural products from terpenes have been reported, including triterpenes, diterpenes, monoterpenes, hemiterpenes, and sesquiterpenes, and more compounds are still under study for their bioactivity [112]. This group of natural compounds is biosynthesized via the methylerythritol phosphate (EMP) and mevalonate pathways [113]. Terpenes play several important roles in plants, and these include their role as signaling molecules for the attraction of insects during pollination and plants’ defense against both abiotic and biotic stress [114]. The isopentenyl diphosphate from the MVA pathway leads to the synthesis of sterols, brassinosteroids, sesquiterpenes, and polyphenols, while the MEP pathway involves the synthesis of phytol, diterpenes, tocopherol, and phytohormones (abscisic acid and gibberellins) [115]. The biosynthesis of the terpenes provides signal molecule compounds with roles in plant defense against abiotic stress [116]. Plant terpenes are responsible for deterring herbivores and attracting insects during pollination by producing a strong odor. More so, PGPR is well characterized for plant protection; the induction of terpenes in plants by PGPR can be beneficial to improve yield and growth in crops [117]. For example, the yield in apple, tomato, and pepper was increased by the use of strains from *Bacillus* and *Pseudomonas* [118].

Phenylpropanoids are natural phenolic compounds with a biosynthesis pathway that involves important secondary metabolites such as anthocyanins, flavones, stilbene, tannins, lignin, flavonoids, and phenolic volatiles, which are synthesized for plant defense against various biotic and abiotic stresses [119]. The metabolic pathway of phenylpropanoids is catalyzed by the enzyme chalcone synthase (CHS), which is involved in the synthesis of different flavonoids by the condensation of malonyl-CoA and p-coumarl-CoA. Phenylpropanoids emitted by microorganisms trigger redox reaction in soils and influence enzymatic activity and also lead to the availability of phytonutrients and hormonal balance. Furthermore, the phenylpropanoid pathway accumulates lignin, which plays a crucial role in thickening lignin layers in plants’ cell walls using reinforced defense structures. The production of flavonoid metabolites plays an important role in plants, such as protecting the plant against heat, UV light, herbivore attack, and pathogen attack [120]. Three critical enzymes in the phenylpropanoid pathway, namely: 4-coumarate-CoA ligase (4CL), Phenylalanine ammonia-lyase (PAL), and cinnamate-4-hydroxylase (C4H), are also enzymes that catalyze phenolic synthesis [121]. Phenolics help produce a number of compounds that help plants recognize potential pathogens, inhibit certain enzymes, attract pollinators, repel herbivores, eliminate competition between two plants, and absorb UV light [122]. A large number of phenolic compounds are found in the bark of conifers where they function as antifungal agents halting the growth of invading organisms by inhibiting their hydrolytic enzymes to reduce their nutritional value for the invading organism [123]. Polyphenolic compounds convey antioxidant activities, and during an attack, are converted to soluble phenolic compounds to add to the defense mechanism [122]. The decrease in the production of phenolics and other compounds from plants during biotic and abiotic stresses leads to the use of microorganisms. Microorganisms in different root locations break down phenolic compounds for the mineralization of soil nitrogen [124]. In the rhizosphere, the phenolics move through and bind to soil matter and are metabolized by the bacterial flora of the soil. The metabolized phenolics then provide an active site, soil porosity, and bioavailability of elements such as copper, iron, boron, zinc, manganese, molybdenum, potassium, and magnesium for plant roots [125].

It was shown that inoculation of cadmium-stressed tree plants with mycorrhiza was followed by increased secondary metabolites such as phenolics, 3-4 dihydroxybenzoic acid, *p*-coumaric acid, and ferulic acid [126]. Yasmin, et al. [127] showed that inoculating soybean (*Glycine max*) plants with plant-associated bacteria increased phenolic content and alleviated heavy metal stress. Some of the secondary metabolites used by microbes to scavenge harmful ROS and alleviate abiotic stress include flavonoids and lignin precursors, phytoalexins, phenylpropanoids, and carotenoids [102]. Microbial-induced secondary metabolites have also been implicated in frost hardiness, drought resistance, heat acclimation, and freezing tolerance in plants [17]. Secondary metabolites do not only scavenge ROS, but they also stimulate chemo-attraction of *Rhizobia* and fungi towards roots so as to establish symbiosis [128]. In support of this view, Sebastiana and colleagues (2014) showed that root colonization with fungi upregulated genes involved in secondary metabolites biosynthesis that provided a means of increasing contact sites and niches for hosting the colonizing hyphae [129]. Figure 2 depicts a proposed model of PGPR and AM against abiotic stress in plants:

The release of phytohormones such as ethylene (ET), auxin, abscisic acid, cytokinin (CKs), and gibberellins (GAs) is known to contribute to plant growth and nutrient availability [130]. Indole-3-acetic acid (IAA) phytohormone, commonly known as auxin, is a key regulator of plant growth and development. Auxin regulates many biological processes such as embryogenesis, vascular tissue differentiation, fruit development, apical dominance, developing seeds, ethylene production, lateral root development, sex expression, and ion fluxes [131]. Ethylene (ET), a gaseous hormone produced from 1-aminocyclopropane-1-carboxylic acid (ACC), also elicits several functions such as germination stimulation, leaf and flower senescence, fruit maturation, adaptation to stress conditions, and resistance to pathogen infection [132]. The production of ET is tissue-specific, and its level increases during biotic and abiotic stress and plays a role in response to herbivores insects and necrotrophic pathogens [133]. Gibberellins (GAs) are classic plant hormones that belong to the tetracyclic diterpenoid family and play a significant role in germination, promoting flowering, stimulating plant elongation, pollen development, and release of seed dormancy [134]. Bacterial endophyte-producing GAs are important in improving seed growth and plant physiology. The *Bacillus amyloliquefaciens* RWL-1 emit GAs in seed-borne *Oryza sativa* to regulate its endogenous phytohormones. The secreted GAs from RWL-1 colonize rice roots and improve plant growth, which shows that isolated RWL-1 could be used as microbial base fertilizer to enhance crop production [135].

## 5. Release of Volatiles and Cadaverine Compounds by Microorganisms to Mitigate Abiotic Stress

Microorganisms from plant roots are versatile in solubilizing, mobilizing, and transforming nutrients when compared with soil-producing bulk [8]. Microorganisms secrete volatile organic compounds (VOCs) that have a beneficial effect on plant growth and development [136]. VOCs occur in lipophilic nature as a complex mixture with low molecular weight (<300 g mol^−1^), high vapor pressure, and low boiling point. The lipophilic compounds modulate physiological processes and travel through the porous soil, liquid, and air [137]. Studies involving the role of microbial VOCs on plant growth are well-documented [22]. The first such study was initiated in 2003, whereby Ryu and colleagues reported a ∼5-fold increase in the *A. thaliana* leaf area due to volatiles released by *B. subtilis* GB03 after 10 days of exposure [2]. Emitted volatile compounds by bacterial species from *Serratia, Pandoraea, Chromobacterium,* and *Burkholderia* genera have been shown to increase the biomass (∼125–620%) of *A. thaliana* [138].

Microorganisms produce diverse volatile metabolites, but the most emitted volatile compounds are propanoic acid, 5-hydroxy-methyl-furfural, butanoic acid, geosmin, camphene, β-caryophyllene, camphor, acetaldehyde, furfural, 2-methylisoborneol, α-pinene, and methanol [139]; 2-heptanol, 2-undecanone, 4-heptanone, 2-pentadecanone, 2-pentanone, and 2- tridecanone are commonly known microbial VOCs [135], while sodorifen, a bicyclic oligomethyl octadiene is a well-known compound produced by *Serratia odorifera* [140]. Two other bioactive compounds are ammonia and dimethyl disulfide (DMDS). The DMDS emitted by *Bacillus cereus* strain has shown to protect corn (Zeamays) and tobacco (*Nicotiana tabacum*) plants against *Cochliobolus heterostrophus* and *Botrytiscinereae*, respectively [141]. The *Cladosporium* sp. and *Ampelomyces* spp. emit major active volatile compounds (methylbenzoate and m-Cresol) in fungal species, which provides ISR defense in *Arabidopsis* against the pathogen *Pseudomonas syringae* pv. tomato DC3000 [142]. 

Polyamines (PAs) are organic polycations that promote plants’ productivity, fruit ripening, and flowering, as well as play a significant part in the management of plant stresses [143]. In plants, cadaverine (Cad), putrescine (Put), spermine (Spm), and spermidine (Spd) are major forms of polyamines that contribute to plant growth and development, and enhanced abiotic stress tolerance to various environmental stresses [144]. Cadaverine is a diamine produced through several biochemical pathways and uses lysine as the main precursor, which is decarboxylated by the enzyme lysine decarboxylase (LDC) [145]. Cadaverine, previously known as the decomposed lysine organic matter found in the environment, has been found to contribute to healthy crop production by maintaining plant growth and development, stress response, cell signaling, and insect defense [146]. Several plants such as rice, barley, wheat, oat, maize, sorghum, and legumes have been shown to produce cadaverine [147]. It is important to note that about 240 different bacteria-species-producing cadaverine have been isolated from spinach leaves [148]. Ali and Khan [13] reported that cadaverine produced from *A. brasilense* Az39 increased root growth and osmotic intolerance in rice seedlings. Several bacteria have been shown to produce cadaverine, which plays a role in oxidative stress, insect defense, the stress response to salt, drought, and heat stressors [148]. Haneburger et al. reported the role of cadaverine produced by *Escherichia coli* in acid stress mediation [149]. Spermidine and putrescine have been reported to produce γ-Aminobutyric acid (GABA) under stress conditions. GABA is nonprotein amino acids conserved in plants for stress responses; its metabolism was postulated to play a role in maintaining carbon/nitrogen, redox regulation, herbivore deterrence, energy production, and pH regulation [150]. The protective role of polyamines has been detected in pigeon pea (*Cajanus cajan*) and mung bean (*Vigna radiata*) during high-temperature stress, whereby Put and Spm enhanced plant protection by scavenging ROS during drought stress, thus improving leaf and root growth, photosynthesis transpiration, and reproductive development [151].

## 6. Conclusions

As the need to feed a growing population increases, along with the chronic demand for global food security, environmental stressors continue to decrease crop production. These stresses have not only decreased crop production but have also caused huge economic losses in the agricultural sector worldwide. To address these effects, strategies ranging from the molecular level, with the isolation and transfer of tolerant genes to improve crop growth to the whole-plant level with conventional breeding, have been developed. With challenges in these approaches, a simple low-cost approach of using microorganisms has been viewed as a promising broad-spectrum means of producing the much-needed food. These microbes have not only improved the growth of stressed plants but have also offered protection against oxidative stress using secondary metabolites. A huge number of potential microorganisms have now been commercialized for use against abiotic stress. Since nitrogen is a source of nutrients for plants, most commercialized microbial fertilizers are products of nitrogen-fixing organisms such as *Azospirillum* spp., *Azotobacter* spp., Actinorhizobium spp., and Rhizobium spp. This has been attributed to their ease of handling and endospore-forming ability that facilitates efficient colonization. An understanding of the mechanisms used by microorganisms against abiotic stress (see Figure 2) may provide new avenues to increase yield beyond conventional breeding and biotechnology.
